# Lithium-ion attack on yttrium oxide in the presence of copper powder during Li plating in a super-concentrated electrolyte†

**DOI:** 10.1039/d0ra10388h

**Published:** 2021-02-03

**Authors:** Tohru Shiga, Yumi Masuoka, Hiroshi Nozaki, Nobuko Ohba

**Affiliations:** Toyota Central Research & Development Laboratories Inc. Yokomichi Nagakute-city Aichi-ken 480-1192 Japan e0560@mosk.tytlabs.co.jp

## Abstract

Li plating/stripping on Cu and Y_2_O_3_ (Cu + Y_2_O_3_) electrodes was examined in a super-concentrated electrolyte of lithium bis(fluorosulfonyl)amide and methylphenylamino-di(trifluoroethyl) phosphate. In principle, Li^+^ ions cannot intercalate into a Y_2_O_3_ crystal because its intercalation potential obtained from first-principles calculations is −1.02 V *vs.* Li^+^/Li. However, a drastic decrease in the electrode potential and a subsequent constant-potential region were observed during Li plating onto a Cu + Y_2_O_3_ electrode, suggesting that Li^+^ interacted with Y_2_O_3_. X-ray diffraction (XRD) patterns and X-ray absorption fine structure (XAFS) spectra of the Cu + Y_2_O_3_ electrodes after the Li plating were recorded to verify this phenomenon. The XRD and XAFS results indicated that the crystallinity of Y_2_O_3_ crystals was lowered because of attack by Li^+^ ions or that the Y_2_O_3_ crystal structure was broken while the +3 valence state of Y was maintained.

## Abstract 2

When Li plating was conducted on a Cu + Y_2_O_3_ composite electrode in a super-concentrated electrolyte, a potential drop and subsequent potential plateau were observed, where Li plating occurs and Li^+^ attacks the Y_2_O_3_ crystals.

## Graphical abstract

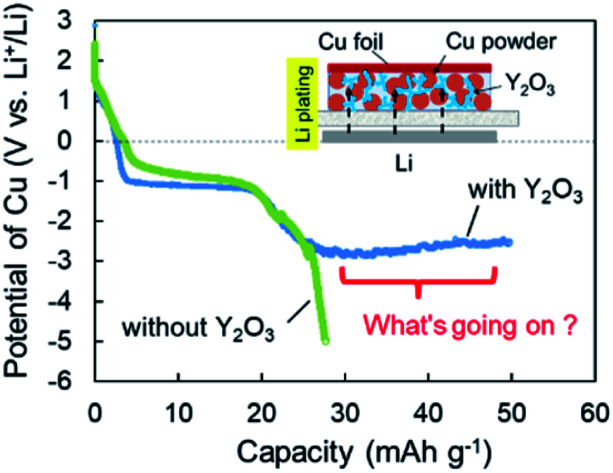

## Introduction

Li^+^-ion batteries (LIBs) are one of the most important rechargeable power devices and are essential to hybrid and electric vehicles. Conventional LIBs composed of lithium cobalt oxide (LiCoO_2_) and graphite operate at 3.7 V. One of the challenges in the development of next-generation battery technologies is increasing the cell voltage. The use of lithium transition-metal oxides such as LiNi_0.5_Mn_1.5_O_4_,^1–3^ LiCoPO_4_,^4–6^ or LiCoMnO_4_ (ref. 7–9) instead of LiCoO_2_ as a cathode material results in high-voltage cells with an operating voltage of ∼5 V. By contrast, for the anode active material, only Li metal can be used to increase the cell voltage. Therefore, research into active materials that can exhibit a redox couple at potentials lower than the Li plating/stripping standard level is a challenge in the development of battery technologies.

Numerous metal oxides have been investigated as anode materials.^10^ For example, niobium(v) oxide (Nb_2_O_5_) exhibits oxidation/reduction behavior expressed by the equation Nb_2_O_5_ + 2Li^+^ + 2e^−^ ⇌ Li_2_Nb_2_O_5_ at potentials between 2.2 V and 1.6 V *vs.* Li^+^/Li. The valence of Nb changes from +5 to +4 during Li^+^ intercalation.^11,12^ Thus, for metal oxides to be used as anode materials, they must have more than one valence. However, yttrium oxide (Y_2_O_3_) has only one valence of +3.^13,14^ If Li^+^ ions are intercalated into a Y_2_O_3_ crystal, either yttrium ions will be reduced to the metal state or oxygen ions will be oxidized. First-principles calculations show that the intercalation potential of Li^+^ into Y_2_O_3_ is −1.02 V (Section 3.2). Therefore, Y_2_O_3_ is theoretically inactive toward Li^+^ insertion; Li electrodeposition occurs preferentially.

**Fig. 1:**
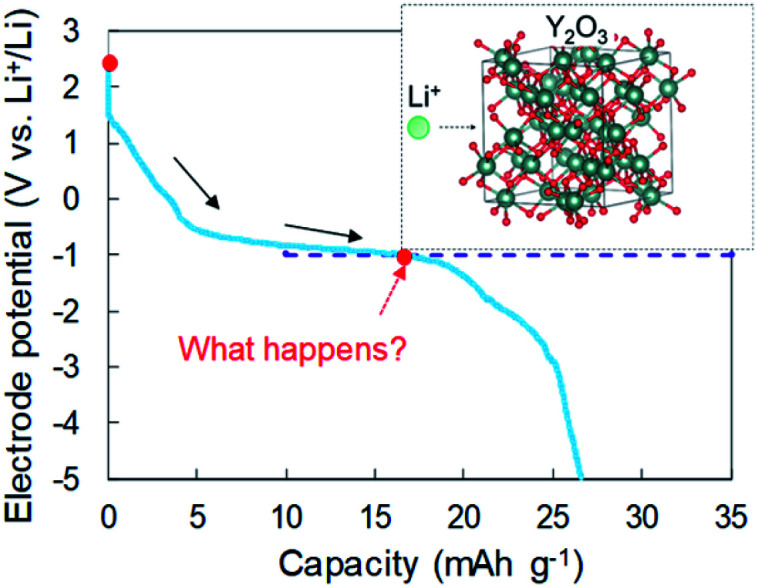
Schematic of the interaction between a Li^+^ ion and a Y_2_O_3_ crystallite.

In our previous reports,^15,16^ we examined Li intercalation into graphite in some super-concentrated electrolytes of lithium bis(fluorosulfonyl)amide (LiFSA) and self-extinguishing solvents. Li plating/stripping has also been investigated in this electrolyte using an electrochemical cell with Cu and Li metal electrodes. A decrease in the potential of the Cu electrode because of solid electrolyte interphase (SEI) formation was observed at the initial stage of the Li plating test. After the SEI had formed, a constant-potential region due to Li electrodeposition appeared. In the later stage of Li plating, a sudden decrease of the Cu electrode potential was observed (Fig. 1). This phenomenon was caused by a large increase in interfacial resistance between the electrolyte and the Cu electrode. When a similar large decrease in potential occurs in a Cu + Y_2_O_3_ composite electrode, Li^+^ ions are speculated to interact with Y_2_O_3_. The Li^+^ intercalation should be observed at −1 V *vs.* Li^+^/Li if the process occurs as planned (blue dotted line in Fig. 1). In the present paper, we prepared a composite electrode of Cu and Y_2_O_3_ powders and examined whether Li^+^ ions insert into the Y_2_O_3_ crystallites.

## Experimental

### Materials

Cu powder was obtained from Kojundo Chemical (average diameter: 1 μm). Y_2_O_3_ nanopowder was purchased from Sigma-Aldrich (average diameter: <100 nm). LiFSA as a Li^+^ salt and methylphenylamino-di(trifluoroethyl) phosphate (PNMePh) as a solvent were obtained from Kishida Chemicals and Tosoh Finechem Corp., respectively. LiFSA was used after drying at 150 °C. The water content in the PNMePh liquid was 37 ppm. Vinylene carbonate (VC), an electrolyte additive,^17,18^ was purchased from Kishida Chemicals (battery grade). The chemical structure of the three compounds was displayed in the ESI (Fig. S1†). The X-ray diffraction (XRD) pattern for the Y_2_O_3_ powder is shown in Fig. S2.†

### Electrochemical measurements

The Cu + Y_2_O_3_ composite electrode was prepared as follows. The Cu and Y_2_O_3_ powders were mixed with polyvinylidene difluoride (PVdF, #9350, Kureha) and *N*-methyl pyrrolidone (NMP; Wako Chemicals) using a kneading machine (ARE-310, Thinky Co.). The mixing speed and time were 2200 rpm and 5 min, respectively. The electrode slurry was spread onto a Cu current collector (20 μm thick) using a doctor-blade technique and dried at 150 °C under vacuum for 3 h. The final mixing ratio of Cu, Y_2_O_3_, and PVdF was 89% : 8.5% : 2.5% by weight.

The Cu + Y_2_O_3_ electrode sheet was pressed to obtain a 14 mm diameter disk electrode with a thickness of 55 μm (Fig. S3†). The loadings of the Cu and Y_2_O_3_ powders were 28–31 mg cm^−2^. A half coin cell (Fig. S4†) was fabricated using the 14 mm diameter disk electrode, two filter papers (200 μm thick, ADVANTEC, 5C), a Li metal disk 18 mm in diameter and 0.4 mm thick (Honjo Metal), and a super-concentrated electrolyte. The super-concentrated electrolyte was prepared by mixing LiFSA and PNMePh in a molar ratio of 1 : 3. The cells were fabricated using a dilute electrolyte with the molar ratio of 1 : 8, and 1 mol L^−1^ LiPF_6_ in a mixture of ethylene carbonate (EC) and dimethyl carbonate (DMC) (Kishida Chemicals, battery grade) in the same manner. The half coin cell was cycled with a charge–discharge apparatus (Hokuto Denko, HJ1001SM8A). The charge (Li plating) was carried out under a current of 0.05 mA. The Li plating/stripping test was also performed using an electrolyte based on VC at a concentration of 1 vol%. Charging was started under a constant current mode at 0.05 mA for 24 h, and the discharge (Li stripping) to 2.0 V was conducted in constant-current mode at 0.05 mA.

### Cyclic voltammetry (CV)

The half coin cells were cycled using a potentiometer (IVIUM Technologies, IVIUMSTST-XR) to obtain CV profiles of Li plating/stripping and Li intercalation in the highly concentrated electrolyte. A Li-metal anode was used as the counter and reference electrodes. CV of the electrolytes was conducted under a sweep rate of 0.2 mV s^−1^ at 25 °C. The CV curves for the super-concentrated electrolyte are displayed in Fig. S5.†

### Electrochemical impedance spectroscopy (EIS)

The Cu + Y_2_O_3_ electrode in the coin cell was used as a working electrode, and a Li-metal anode was used as the counter and reference electrodes. EIS was conducted at 25 °C in the frequency range from 7 MHz to 20 mHz with a BioLogic SP-300 potentiostat to investigate the formation of a passive layer on the Cu + Y_2_O_3_ electrode. It was also conducted between 25 and 51 °C with the amplitude of the sinusoidal potential adjusted in 10 mV increments. Impedance spectra were fit using the EC-Lab Zfit software.

### Analysis

The Cu + Y_2_O_3_ electrodes before and after Li plating/stripping were analyzed by XRD (Rigaku, Ultima IV). The Cu + Y_2_O_3_ electrode sample that contained the electrolyte was affixed to a glassy cell holder using Kapton tape in an Ar-filled glove box. The XRD profile was recorded in the 2*θ* range from 10 to 80° at a sweeping rate of 10° min^−1^. The morphology of Li plating in the sample was examined by field-emission scanning electron microscopy (FE-SEM, JEOL, JSM-7000F). The Cu + Y_2_O_3_ electrode was rinsed with DMC three times in an Ar-filled glove box and dried under vacuum. Energy-dispersive X-ray spectroscopy was used to identify passivation films on the Cu, electrodeposited Li, and Y_2_O_3_. ^7^Li nuclear magnetic resonance (NMR) spectroscopy was used to examine Li compounds after the Li plating tests; measurements were performed using a Bruker NMR spectrometer (AVANCE 400). In an argon-filled glove box, the Cu and Y_2_O_3_ powders were stripped from Cu foil and washed several times with DMC. After drying under vacuum, the powders were packed into a ZrO_2_ tube. The NMR reference material was 1 mol L^−1^ LiCl aqueous solution. The measurement conditions are summarized in Table S1.† X-ray absorption fine structure (XAFS) analysis of the Y_2_O_3_ powder after the Li plating test was conducted through-the-law to identify the valence of Y after the Li plating. In an Ar-filled glove box, the Cu and Y_2_O_3_ powders were stripped from Cu foil, washed with DMC several times, and dried under vacuum. XAFS measurements were carried out at the Toray Research Center Inc (Table S2†).

### Computational details

First-principles calculations were performed for cubic Y_2_O_3_ crystals using the Vienna *Ab initio* Simulation Package (VASP)^19^ with the generalized gradient approximation of Perdew–Burke–Ernzerhof (PBE)^20^ for the exchange-correlation terms in the density functional theory and using the projector-augmented wave (PAW) method^21^ for describing the ion–electron interaction. The cutoff energy for the plane-wave expansion was set to 500 eV. The spin polarization was considered in all calculations because the total number of electrons may become odd upon Li insertion. The insertion sites of Li were estimated using the bond valence sum (BVS),^22^ which is one of the indices used to evaluate the stability of atoms (ions) in a crystal structure. Because the valence of the Li^+^ ion in the oxide was assumed to be +1, we inserted Li^+^ ions at sites with a BVS close to 1.

## Results and discussion

### Observation of potential drop during Li plating

Li plating/stripping was studied in each of the investigated electrolytes. In general, the SEI forms at the initial stage of Li plating, and it follows the electrodeposition of Li while a constant voltage is maintained. Electrical shorts due to Li dendrite growth were detected at the end of Li plating. The first Li plating test in this study was conducted in the super-concentrated electrolyte using an electrode fabricated with only Cu powder (*i.e.*, without Y_2_O_3_). Its potential–capacity curve during the Li plating process is presented in the ESI (Fig. S6†). The horizontal axis unit in Fig. S6† is electrical capacity per weight of Cu powder. The initial stage between A and B reflects SEI formation due to reductive decomposition of the PNMePh solvent. The region from B to C corresponds only to Li plating onto Cu powder and Cu foil. A large potential drop was unexpectedly observed when the capacity exceeded 25 mA h g^−1^. The potential finally arrived at −5 V (point D). To determine the cause of the potential drop, we conducted EIS measurements corresponding to points A–D in the potential–capacity curve.

**Fig. 2:**
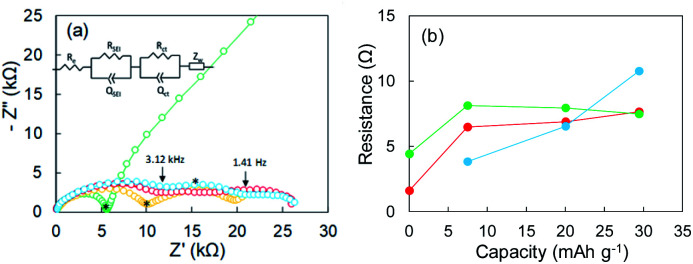
(a) Nyquist plots of a Cu powder electrode before Li plating (green) and after 7.5 mA h g^−1^ (yellow), 20.1 mA h g^−1^ (red), and 29.4 mA h g^−1^ (blue) of Li plating. (b) Relation between resistance and Li plating capacity; *R*_SEI_ (red), *R*_ct_ (green), and *R*_ct-c_ (blue). The asterisks in subfigure (a) represent portions corresponding to 667 Hz.

EIS is a powerful method for obtaining information about the electrochemical behavior at electrode–electrolyte interphases. The green line in Fig. 2a represents the Nyquist plot corresponding to point A (pristine) in Fig. S6.† One distorted semicircle is observed in the high-frequency range from 7 MHz to 667 Hz, and the linear portion of *Z*′ and *Z*′′ appears in the low-frequency range. An equivalent circuit composed of *R*_e_ and two parallel units of resistance and capacity (*R*_SEI_|*Q*_SEI_, *R*_ct_|*Q*_ct_) and *Z*_w_ in Fig. 2a was used to analyze impedance spectrum A, where *R*_e_ is the electrolyte resistance, *R*_SEI_ and *Q*_SEI_ are the resistance and constant phase element (capacity)^23,24^ of the SEI on the metallic Li anode at high frequencies, respectively, *R*_ct_ and *Q*_ct_ are the charge-transfer resistance at the Cu electrode–electrolyte interface and the constant phase element for the double-layer capacitance at low frequencies, respectively, and *Z*_w_ is the Warburg impedance.^25,26^ The analysis results for the pristine sample were *R*_e_ = 0.103 kΩ, *R*_SEI_ = 1.596 kΩ (*Q*_SEI_ = 5.3 nF), and *R*_ct_ = 4.421 kΩ (*Q*_ct_ = 15.6 nF). The Nyquist plot changed dramatically as Li was plated. The Nyquist plots corresponding to points B and C are represented by yellow and red lines, respectively, in Fig. 2a. The linear portion of *Z*′ and *Z*′′ for the pristine sample disappeared at frequencies less than 667 Hz, and the second semicircle appeared (yellow line). As Li plating proceeded further, the first semicircle extended horizontally (red line). A third semicircle with a peak at 667 Hz was detected in the medium-frequency range between 3.12 kHz and 1.41 Hz. The sample in which the potential drop occurred exhibited a larger semi-arc at medium frequencies (blue). For the three samples after Li plating (corresponding to points B, C, and D in Fig. S6†), an equivalent circuit comprising three components (*R*_SEI_|*Q*_SEI_, *R*_cu-in_|*Q*_cu-in_, *R*_ct_|*Q*_ct_) was used, where *R*_cu-in_ and *Q*_cu-in_ are the charge-transfer resistance and constant phase element in the Cu powder electrode in the medium-frequency range.^27^ The potential drop was attributed to the SEI on electrodeposited Li in the Cu electrode. The resistances for the three components (*R*_SEI_, *R*_cu-in_, and *R*_ct_) are summarized in Fig. 2b, and the constant phase elements for each component are shown in Fig. S7.† The increase in *R*_cu-in_ with plating capacity *y* is characteristic. It was greater than 10 kΩ at point D. We therefore concluded that a large potential drop was associated with the increase in *R*_cu-in_. The EIS data for the cell fabricated using only Cu foil as an electrode are shown in the ESI (Fig. S8†). Its EIS performance was similar to that of the Cu powder electrode.

### Calculation of Li^+^ insertion potential

The Li^+^ insertion potential (*V*) was estimated from the total energy difference of the reaction Y_2_O_3_ + 1.5Li^+^ + e^−^ → Li_1.5_Y_2_O_3_ by the following equation:^28^
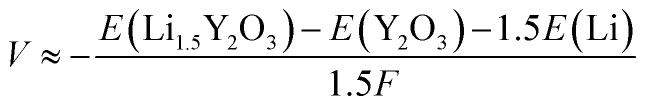
where *F* is Faraday's constant. The valence for all elements in Y_2_O_3_ and Li_1.5_Y_2_O_3_ was estimated by Bader charge analysis^29^ (Table S3†). The crystal structures of bulk Y_2_O_3_ and Li_1.5_Y_2_O_3_ are described in Fig. S9.† The calculated Li^+^ insertion potential was −1.02 V *vs.* Li^+^/Li.

### Li plating test and XRD analysis

**Fig. 3:**
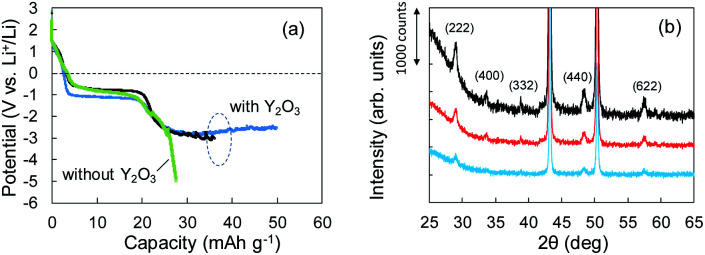
(a) Potential–capacity curves for Li plating tests without (green) and with (blue, black) Y_2_O_3_. (b) XRD patterns for Cu + Y_2_O_3_ electrodes: pristine (black) and after 36.1 mA h g^−1^ (red) and 49.8 mA h g^−1^ (blue) of Li plating.

According to the concept presented in Fig. 1, as Li plating proceeds, the potential of the Cu electrode begins to drop. If Li^+^ intercalation is assumed to occur, then the potential will soon become constant when it reaches −1.0 V. To verify this idea, Li plating tests were conducted with cells fabricated using Cu powder electrodes with and without Y_2_O_3_ powder. The test results are summarized in Fig. 3a. In the case of the cell without Y_2_O_3_ (green line), the potential of the Cu electrode showed a constant value of −0.6 V; it then decreased slowly when the capacity exceeded 20 mA h g^−1^, reaching −5 V at a capacity of 28 mA h g^−1^. By contrast, the cell with a Cu + Y_2_O_3_ electrode exhibited two potential plateaus; the second plateau was observed at −3 V *vs.* Li^+^/Li, suggesting an interaction between Li^+^ ions and Y_2_O_3_ crystals. The constant-potential region between 26 and 49.6 mA h g^−1^ corresponds to 0.708 mA h of electrical capacity. If Li^+^ insertion follows the reaction Y_2_O_3_ + 1.5Li → Li_1.5_Y_2_O_3_, the calculated insertion capacity is 0.541 mA h (mass of Y_2_O_3_ in the electrode = 3.04 mg; 3.04 mg × 0.001 × 26 800 × 1.5/225.8 = 0.541 mA h). Thus, the experimental and calculated values are similar. In addition, Li plating-stripping (charge–discharge) curves were shown in Fig. S10 and S11.†

**Fig. 4:**
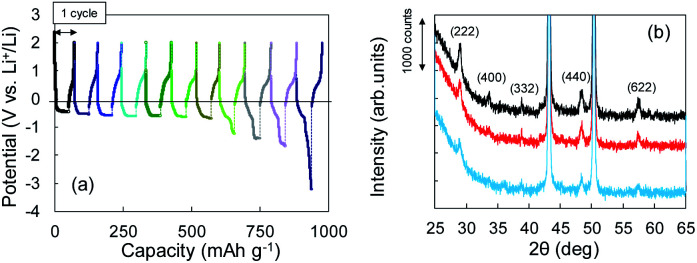
(a) Potential–capacity curves for Li plating/stripping tests. (b) XRD patterns for Cu + Y_2_O_3_ electrodes: pristine (black), after the fifth cycle (red), and after the eleventh cycle (blue).

The Li^+^ insertion may be occurring along with Li plating. To examine the Li^+^ insertion process, we collected XRD patterns of the Cu + Y_2_O_3_ electrodes after Li plating to 36.1 mA h g^−1^ and 49.8 mA h g^−1^ (Fig. 3b). The two sharp XRD peaks at 2*θ* = 43.34° and 50.22° are caused by the Cu powder and Cu foil. Five characteristic signals due to Y_2_O_3_ cubic crystals are observed at 2*θ* = 29.06°, 33.58°, 39.68°, 48.32°, and 57.58° in the XRD pattern of the pristine sample. These signals are due to diffractions from the (222), (400), (332), (440), and (622) planes of cubic Y_2_O_3_ crystals, respectively.^30,31^ As the Li plating proceeded, the intensities of these five peaks decreased. These results suggest that the Y_2_O_3_ crystal size decreased or that the crystal structure was destroyed by the insertion of Li^+^ ions. The Li plating tests clearly show that Li^+^ ions attacked and interacted with Y_2_O_3_ crystals at potentials below the standard Li plating and stripping potentials. To reconfirm this phenomenon, another Li plating/stripping test was conducted in the super-concentrated electrolyte containing VC as an additive. The test with a discharge current of 0.05 mA for 24 h and a charge current of 0.05 mA to 2.0 V was repeated for ten cycles. A potential drop similar to that in Fig. 3a was observed in the discharge step of the seventh cycle (Fig. 4a). XRD patterns of the pristine Cu + Y_2_O_3_ electrodes and those after the fifth cycle and the eleventh cycle are summarized in Fig. 4b. The five characteristic XRD peaks for Y_2_O_3_ disappeared after the tenth cycle. Fig. 4b also shows that the intensity of the XRD signals recovered after Li stripping. Thus, Li^+^ ions were confirmed to have interacted with Y_2_O_3_ crystals at potentials less than the Li plating/stripping potentials.

### XAFS analysis

**Fig. 5:**
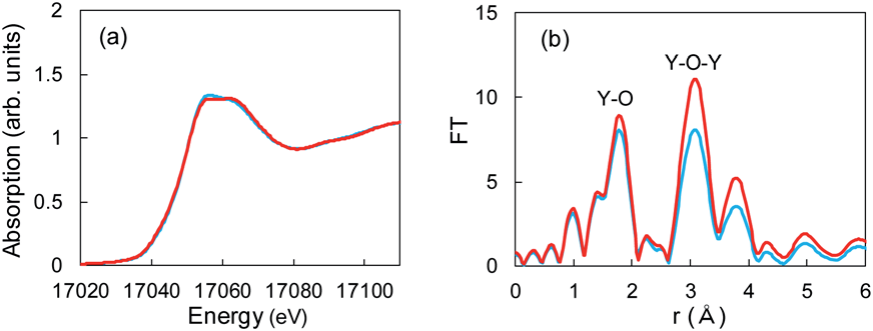
(a) XANES and (b) EXAFS spectra for Y_2_O_3_ powder (red) and the Cu + Y_2_O_3_ electrode after the Li plating test (blue).

The aforementioned XRD studies indicated that Li^+^ ions might have interacted with Y_2_O_3_ crystals during Li plating. If Li^+^-insertion into Y_2_O_3_ occurred, the valence of Y or the atomic distance between and Y and O should have changed. XAFS is a powerful method for characterizing local structures in Y_2_O_3_ crystals. We conducted XAFS measurements for a sample after 55 mA h g^−1^ of Li plating. Fig. 5a shows the Y K-end X-ray absorption near edge structure (XANES) spectra of the sample and Y_2_O_3_ crystal powder. The absorption end of Y_2_O_3_ was 17 040 eV,^32^ and the main absorption peaks were observed in the range from 17 050 to 17 070 eV. In the spectrum of the Cu + Y_2_O_3_ electrode after the Li plating, the absorption end rise was the same as that observed in the spectrum of the Y_2_O_3_ powder. This result shows that similar XANES profiles were obtained for the Cu + Y_2_O_3_ electrode after Li plating and for the Y_2_O_3_ powder even though a slight difference was observed in their main peaks. Therefore, we concluded that the valence of the Y element in the Cu + Y_2_O_3_ electrode was +3.

The extended X-ray absorption fine structure (EXAFS) spectrum is located more than 100 eV from the absorption end, and the Fourier transformation of the real spectrum provides information about the local structure, such as atomic distances. Y K-end FT-EXAFS spectra are displayed in Fig. 5b. The peak of the first close connection, which was associated with Y–O, appeared in the range from 1.2 to 2.0 Å in the spectrum of the Y_2_O_3_ powder. The peak of the second close connection, which was associated with Y–O–Y, was located between 2.7 and 3.4 Å. The EXAFS spectral shape of the Cu + Y_2_O_3_ electrode exhibited the same characteristics as that of the Y_2_O_3_ powder, and the first and second close connections were detected in the same position. Therefore, the coordination environment around Y in the Cu + Y_2_O_3_ electrode was similar that around Y in the Y_2_O_3_ powder. In summary, XANES and EXAFS studies indicated that Li^+^ ions were not inserted into the Y_2_O_3_ crystallites during Li plating.

### FE-SEM images and EDX analysis

**Fig. 6:**
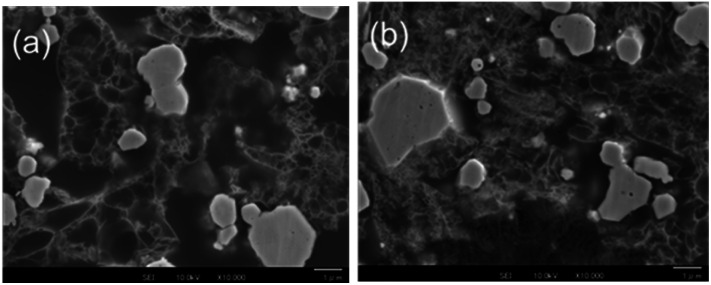
High-resolution FE-SEM images of the cross section of Cu + Y_2_O_3_ electrode (a) before and (b) after Li plating. The magnification is 10 000×, and the scale bar is 1 μm.

The Li electrodeposition and Y_2_O_3_ powder in the Cu + Y_2_O_3_ electrode after the Li plating test to 50 mA h g^−1^ was visualized by FE-SEM. Fig. S12a† shows an FE-SEM image of the cross section of the Cu + Y_2_O_3_ electrode. The microscope magnification was 1000×. Many needle-like crystals, which may be metallic Li, were observed near the Cu foil (yellow dotted ellipses). An enlarged FE-SEM image of the needle-like crystal is shown in Fig. S12b.† FE-SEM images for the Cu + Y_2_O_3_ electrode at 1000× magnification are displayed in Fig. 6a and b to highlight the Y_2_O_3_. The Y_2_O_3_ powders were aggregated, forming a crosslinking mesh with a large hole before the plating. By contrast, the mesh structure after Li plating was broken, indicating that the Y_2_O_3_ powder exerted some influence during the Li plating.

**Fig. 7:**
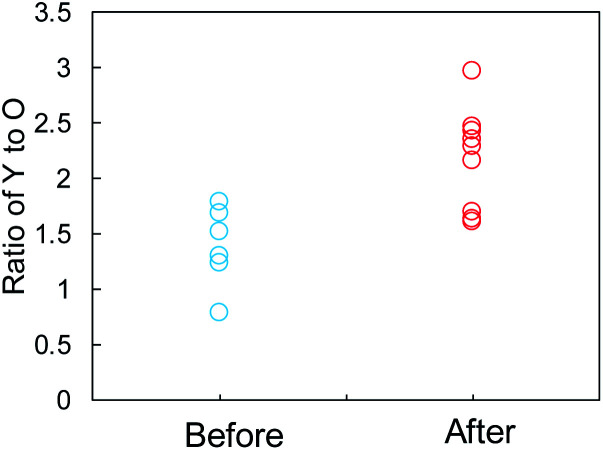
Comparison of the Y-to-O ratio determined by EDX elemental analysis of the Cu + Y_2_O_3_ electrode before and after Li plating.

The SEI films on the Y_2_O_3_ powder, *i.e.*, Li compounds, were further characterized by EDX analysis. We focused on some domains of the Y_2_O_3_ powder with a square of less than 1 μm on one side and recorded the elemental distributions for Y, O, C, F, and Cu. The EDX measurement positions on the sample after Li plating are displayed in Fig. S13a,† and the EDX results are summarized in Fig. S13b.† The EDX results for the sample before Li plating are presented in Fig. S14.†Fig. 7 shows the Y/O ratio for the samples before and after Li plating. The ratio of the sample before the plating varied between 0.8 and 1.8. By contrast, the Y_2_O_3_ domains after the plating were divided into two ranges of 1.6 ≤ Y/O ≤ 1.7 and 2.1 ≤ Y/O ≤ 3.0. The latter ratio reflects the domains after the interaction between Li^+^ and Y_2_O_3_. We cannot currently explain why the ratio increases.

### ^7^Li-NMR measurement

**Fig. 8:**
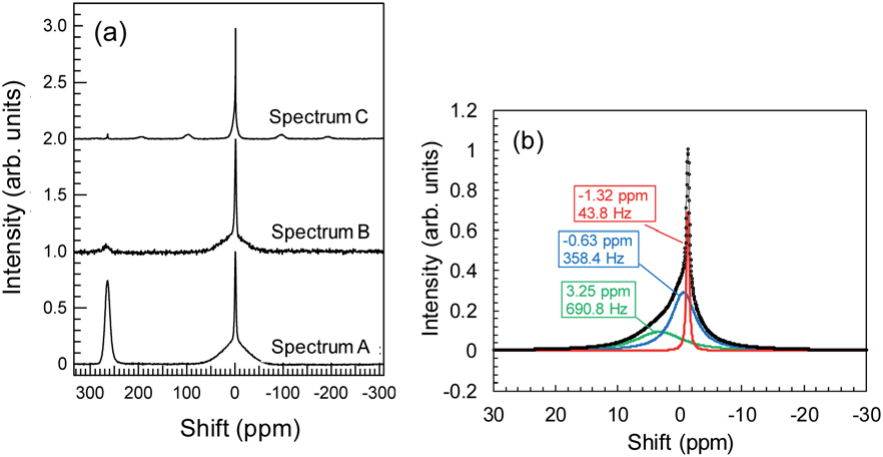
(a) ^7^Li-NMR spectra for Cu + Y_2_O_3_ electrodes with Cu foil (spectrum A), without Cu foil and without rotating (spectrum B), and without Cu foil and with rotating (spectrum C); (b) waveform separation results for spectrum C in the region near 0 ppm.

During Li plating tests, many needle-like crystals grew on the Cu powder and Cu foil. These crystals may be metallic Li. The metallic Li reacts with the electrolyte, resulting in the formation of SEIs at the surface of the Cu components. In the same manner, Li^+^ ions, which interact with Y_2_O_3_, can induce reductive decomposition of the electrolyte. To characterize the deactivated Li, we conducted ^7^Li-NMR measurements. NMR spectrum A in Fig. 8a corresponds to the sample of Cu + Y_2_O_3_ electrode involving Cu foil and LiFSA/PNMePh electrolyte after the Li plating test. A strong peak is observed at 260 ppm, which, according to Grey *et al.*,^33^ is attributable to metallic Li. The broad signal at approximately 0 ppm provided information about the Li^+^ ions in LiFSA and Li compounds resulting from the reductive decomposition of electrolyte by metallic Li.^34–36^ NMR spectra B and C correspond to the Cu + Y_2_O_3_ electrode materials stripped from Cu foil in measurements conducted without and with rotation of the NMR sample tube. After the Cu + Y_2_O_3_ electrode was stripped from the Cu foil, the intensity of the peak due to metallic Li decreased sharply, indicating that the metallic Li existed in the vicinity of Cu foil. Because spectrum C is high-resolution NMR data, we carried out waveform deconvolution for the broad NMR peak at ∼0 ppm. As shown in Fig. 8b, the NMR waveform was deconvoluted into three waves with peaks at −0.63 ppm, −1.32 ppm, or 3.25 ppm. The percentage of each peak was 32%, 14%, and 54%, respectively. The wave with the peak at −1.32 ppm is attributed to Li^+^ ions in the LiFSA/PNMePh electrolyte because it was observed in the spectrum of the pristine sample before the Li plating test (Fig. S15†). The other two waves reflected the reductive decomposition. Letellier *et al.* studied the SEI in LiPF_6_/(EC + DMC) electrolyte using ^7^Li-NMR and identified Li_2_CO_3_, LiF, Li_2_O, LiOH, and ROCO_2_Li as SEI components.^37^ Because the ^7^Li-NMR signal of LiF appears between 0 and −1 ppm, the wave with the peak of −0.63 ppm in this study may be due to an analogue of LiF. The ^7^Li-NMR signal of Li_2_CO_3_ was detected at 3.89 ppm; therefore, the Li compound with a peak at 3.25 ppm is likely an oxide.

## Conclusions

Research into the electrochemical behavior of certain carriers at potentials less than the standard Li^+^/Li plating level is a challenging and unknown technical field and will lead to immensely useful anode materials for batteries. Li^+^ insertion into cubic Y_2_O_3_ crystals is theoretically not possible because the insertion potential is −1.02 V. However, we observed a drastic potential drop of a Cu electrode during Li plating onto Cu in a super-concentrated electrolyte of LiFSA and PNMePh. This unique behavior suggested the possibility of Li^+^ attacking Y_2_O_3_ at a potential of −1.02 V. In this study, Li plating tests on Cu were conducted in the presence of Y_2_O_3_ cubic powder. The Li plating test results and some spectroscopic analyses, including XRD and XAFS, indicate an interaction between Li^+^ and Y_2_O_3_ that leads to a decrease in crystallinity. This unexpected discovery stemmed from our investigation of the decrease in electrode potential in the super-concentrated electrolyte, and we expect similar phenomena to be observed in other highly concentrated electrolyte systems. We speculate that this approach will provide a path for the design of new active materials.

## Conflicts of interest

The authors declare no competing financial interest.

## Supplementary Material

RA-011-D0RA10388H-s001
