# Supplementary material for: Lithium-ion attack on yttrium oxide in the presence of copper powder during Li plating in a super-concentrated electrolyte
Source: RSC Adv. 2021 Feb 3;11(11):6361–6. doi: 10.1039/d0ra10388h (PMC8694813; doi:10.1039/d0ra10388h)
Supplement: RA-011-D0RA10388H-s001 [file RA-011-D0RA10388H-s001.pdf]

Electronic Supplementary Material (ESI) for RSC Advances.
This journal is © The Royal Society of Chemistry 2021

Supplementary information

Lithium-ion attack on yttrium oxide in the presence of copper powder during Li plating in a super-concentrated electrolyte

Tohru Shiga*, Yumi Masuoka, Hiroshi Nozaki, and Nobuko Ohba

Toyota Central Research & Development Laboratories Inc.
Yokomichi, Nagakute-city, Aichi-ken, 480-1192 Japan

*Corresponding author. E-mail: e0560@mosk.tytlabs.co.jp

1. Materials

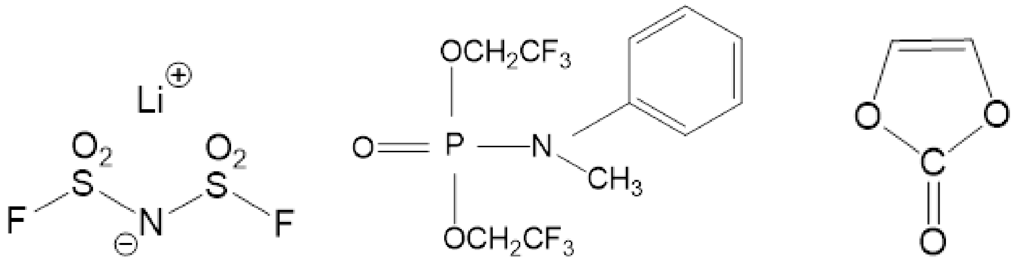

(LiFSA) (PNMePh) (VC)

Figure S1. Chemical structures of LiFSA, PNMePh, and VC

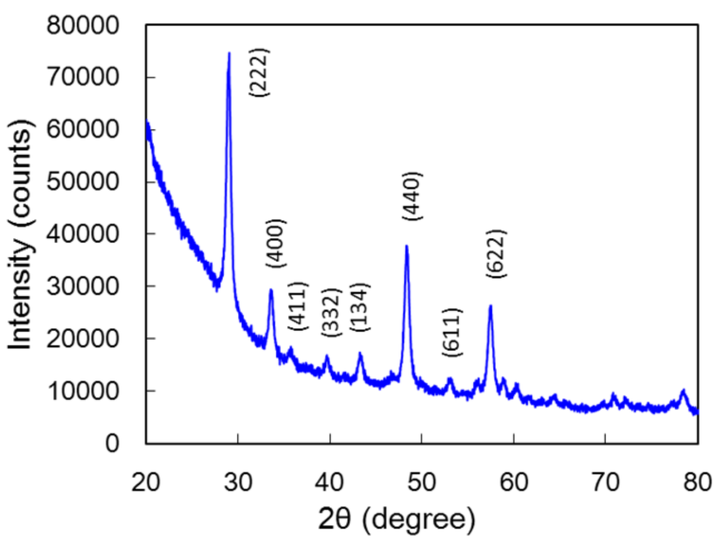

Figure S2. XRD pattern for Y_2O_3 powder.

2. Experimental

2.1. Electrode

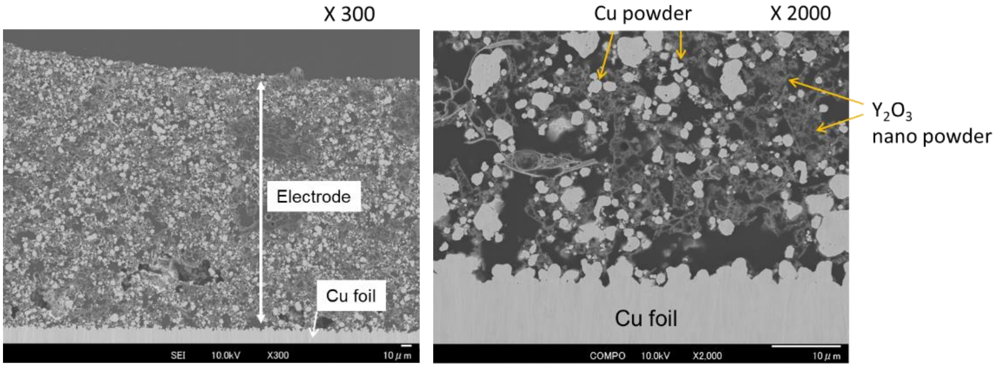

Figure S3. SEM images of the cross section of a Cu+Y_2O_3 electrode.

2.2. Electrochemical cell

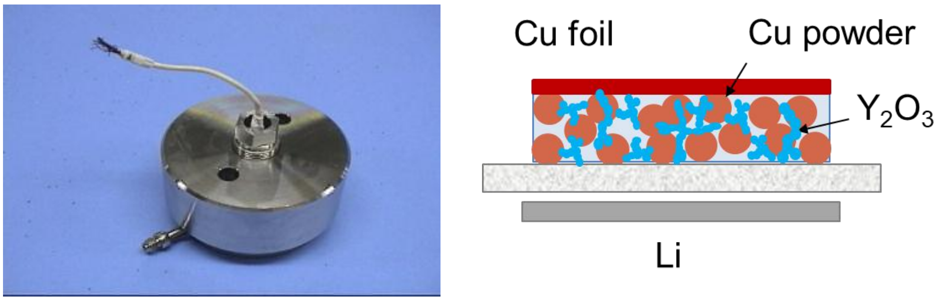

Figure S4. Photograph and schematic of an electrochemical cell.

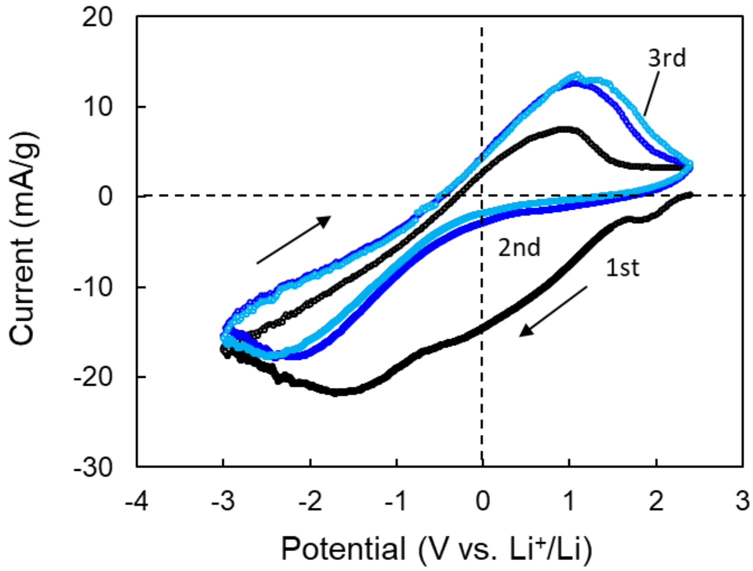

Figure S5. The 1st through 3rd cyclic voltammograms for LiFSA/PNMePh super-concentrated electrolyte. The sweep rate was 1 mV/sec.

Table S1. ^{7}Li-NMR measurement parameters

Measurement frequency	155.5080156 MHz
Spectral width	100 kHz
Pulse width	1.0 μm (30°pulse)
Pulse repetition time	12 sec
Observation point	8192 points
Reference material	1M LiCl aquous solution
Temperature	Room temperature
Rotational frequency	0 Hz, 15 Hz
Sample tube	ZrO_2, inner diameter 1 mm<br>Length 2.5 mm

Table S2. XAFS measurement parameters

Experimental facility	Aichi Synchrotron Radiation Center in Japan
Experimental station	BL11S2
Split	Si(111)2Crystal spectroscopy
Absorption end	Y-K absorption end (17038.0 eV)
Detection method	Through the law
Detectors	Ion chambers

3. Results

3.1. Li plating behavior for the cell fabricated using only Cu powder

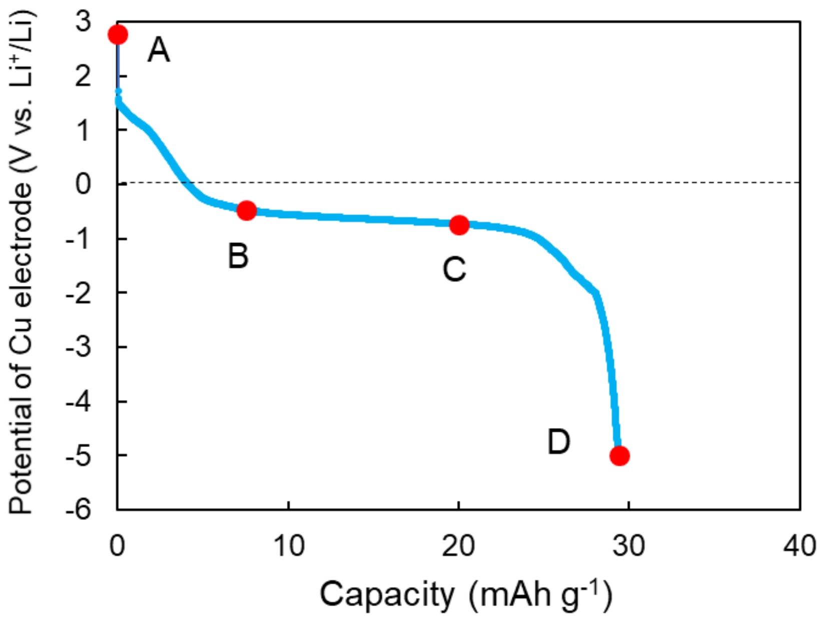

Figure S6. Cu electrode potential-capacity curve for the electrode fabricated using only Cu powder. The samples A,B,C, and D were applied to the EIS measurement.

3.2. EIS results

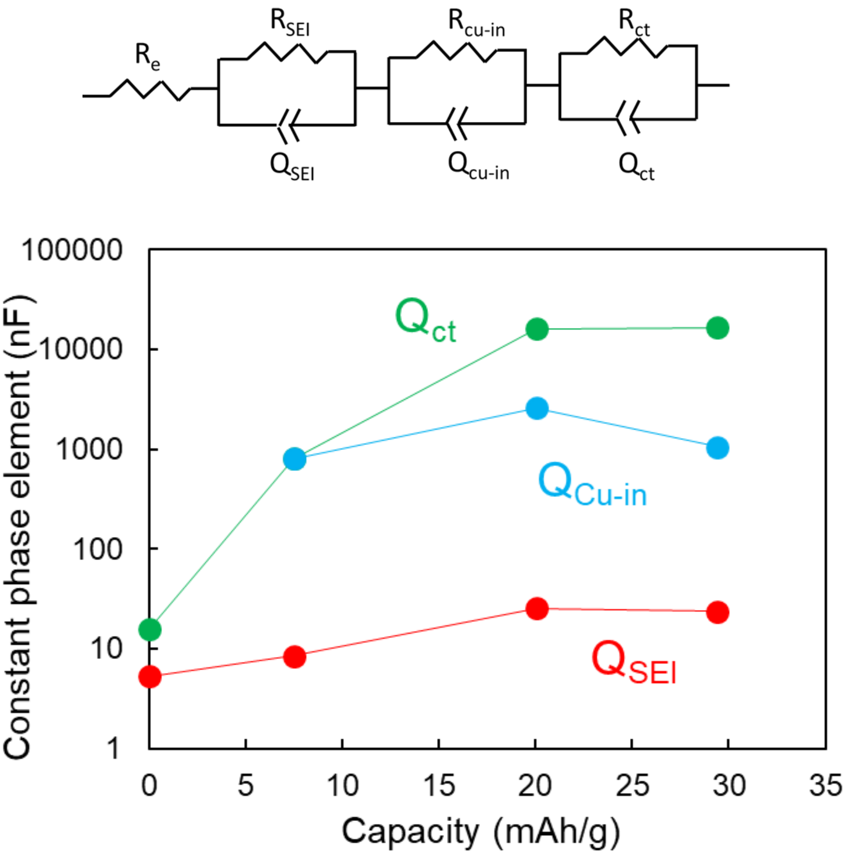

Figure S7. Equivalent circuit (top) and constant phase elements (bottom) for the three components of the cell using Cu powder electrode.

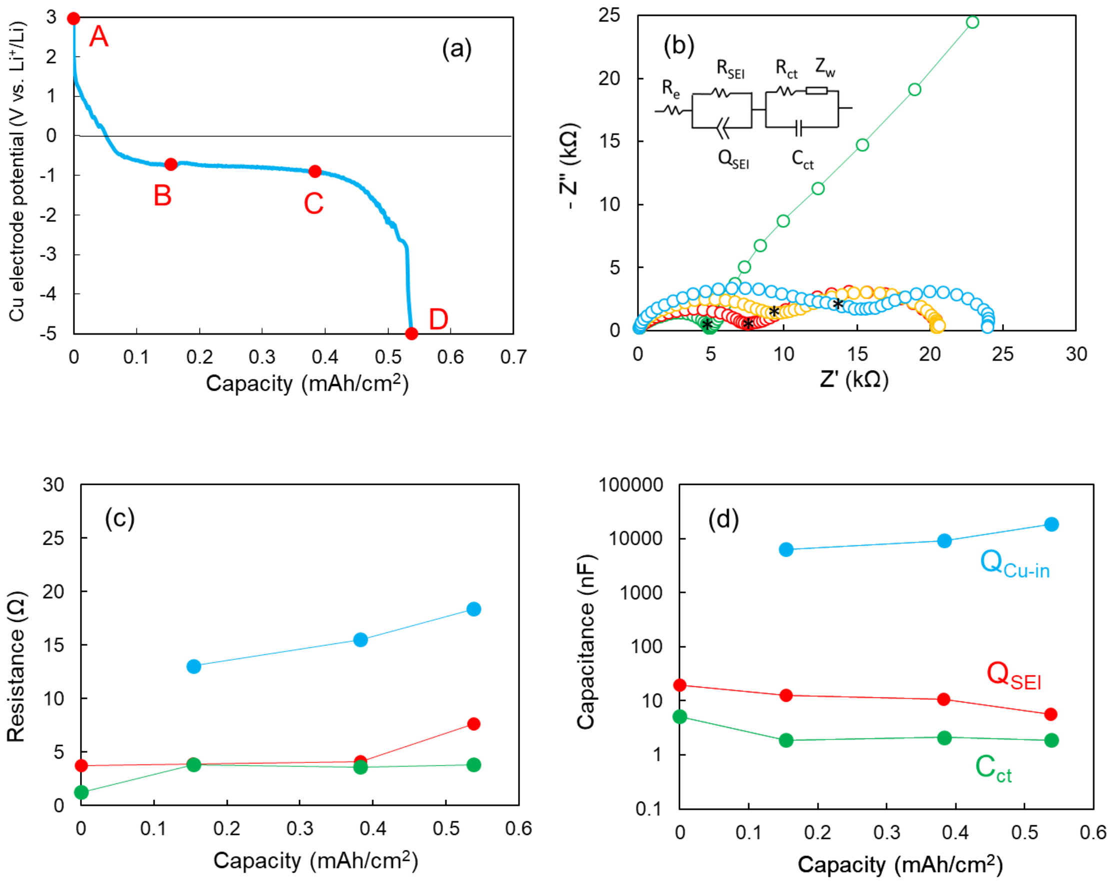

Figure S8. EIS results for the cell using Cu foil electrode.

3.3. Calculation results

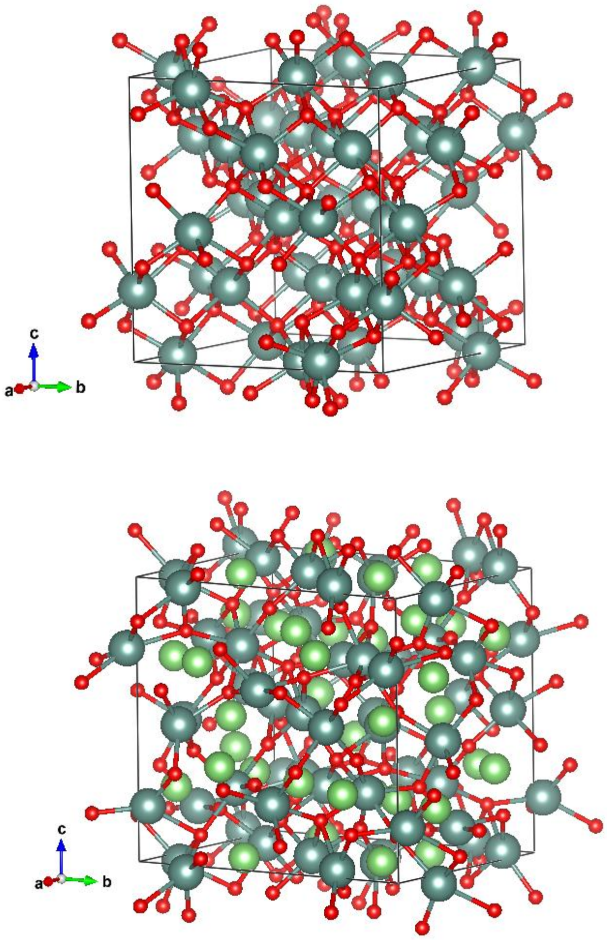

Figure S9. Crystal structure of bulk Y_2O_3 (upper) and $Li_{1.5}Y_2O_3$ (bottom). The dark and light green, and red circles represent **y**ttrium, lithium, and oxygen elements in turn.

Table S3. Bader charge (*e*) for each element in Y_2O_3 and $Li_{1.5}Y_2O_3$

	Y_2O_3	$Li_{1.5}Y_2O_3$
Y	2.14	1.62
O	−1.43	−1.52
Li	−	0.85

3.4. Charge-discharge curves for the Cu+Y_2O_3 cells

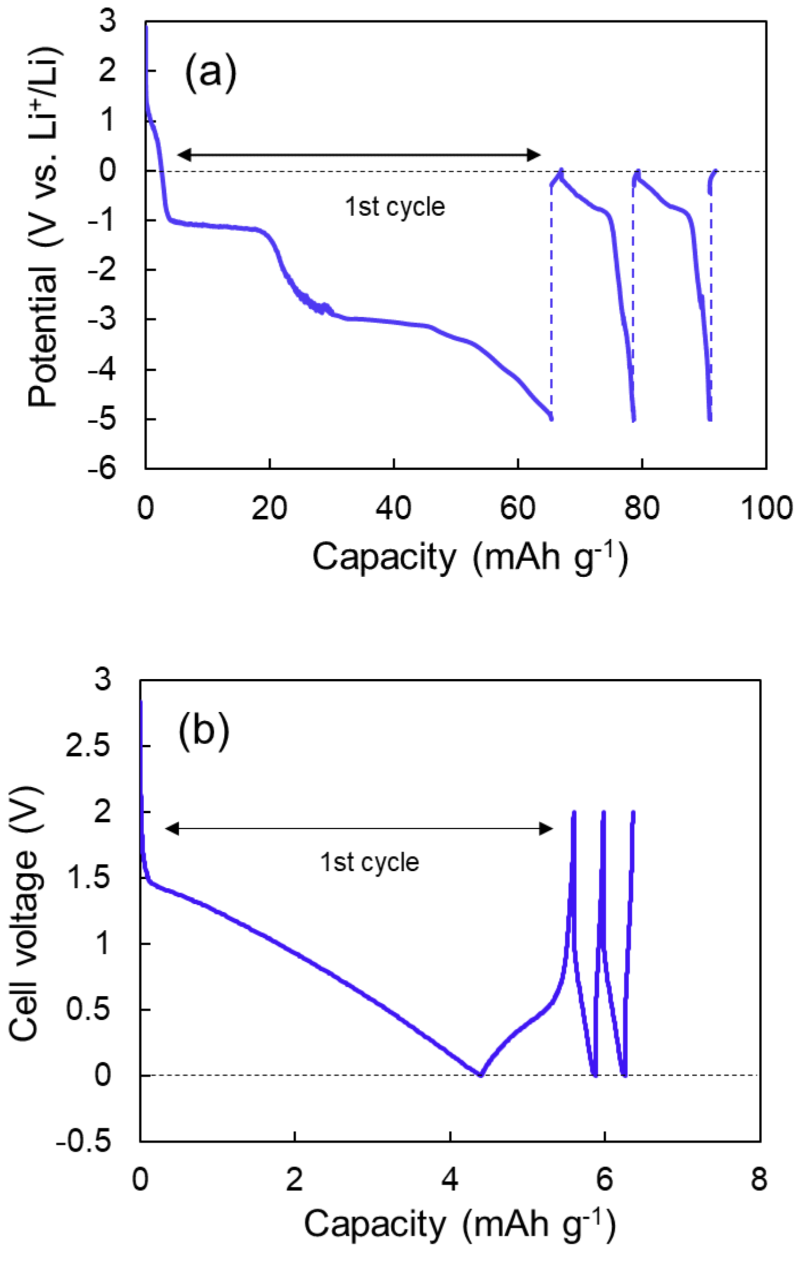

Figure S10. Charge/discharge curves for Cu+Y_2O_3 cells using Li metal electrode between (a) -5 V - 0V, and (b) 0 V - +2 V.

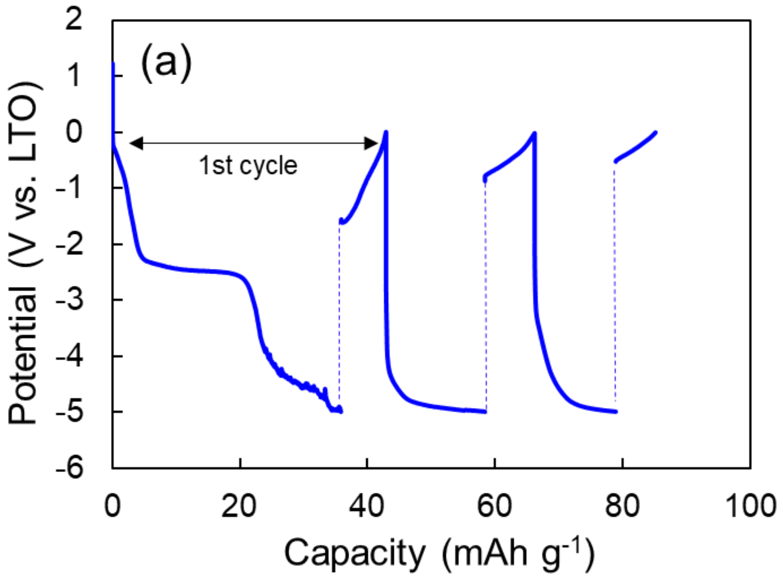

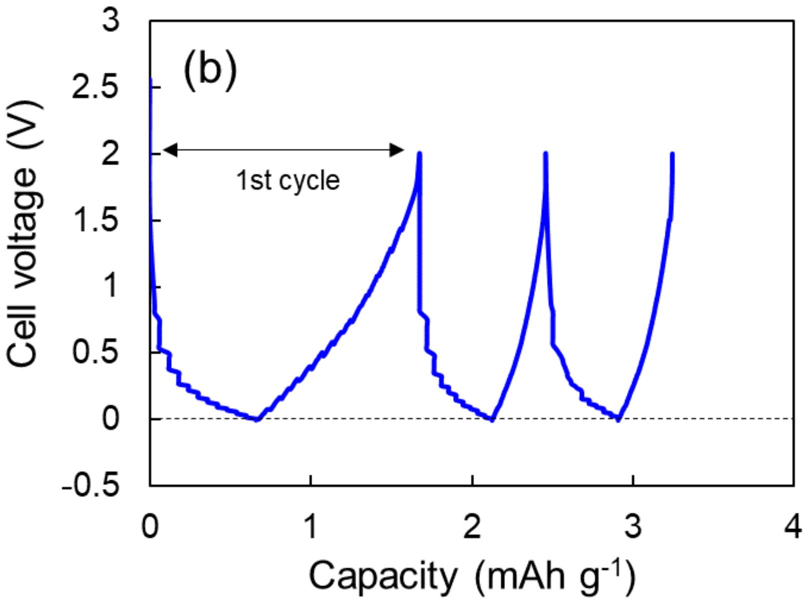

Figure S11. Charge/discharge curves for $Cu+Y_2O_3$ cells using Li-doped LTO ($Li_4Ti_5O_{12}$) electrode between (a) -5 V - 0V, and (b) 0 V - +2V.

3.5. FE-SEM images

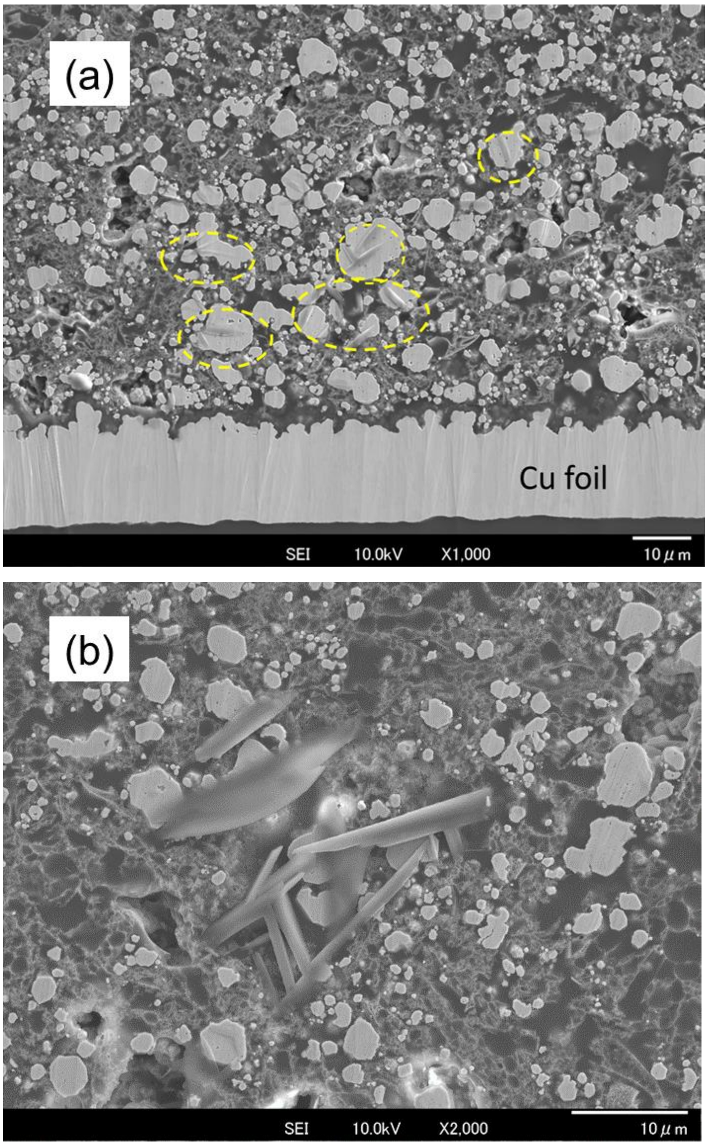

Figure S12. FE-SEM images of electrodeposited metallic lithium at (a) 1000x, and (b) 2000x magnification.

3.6. EDX results

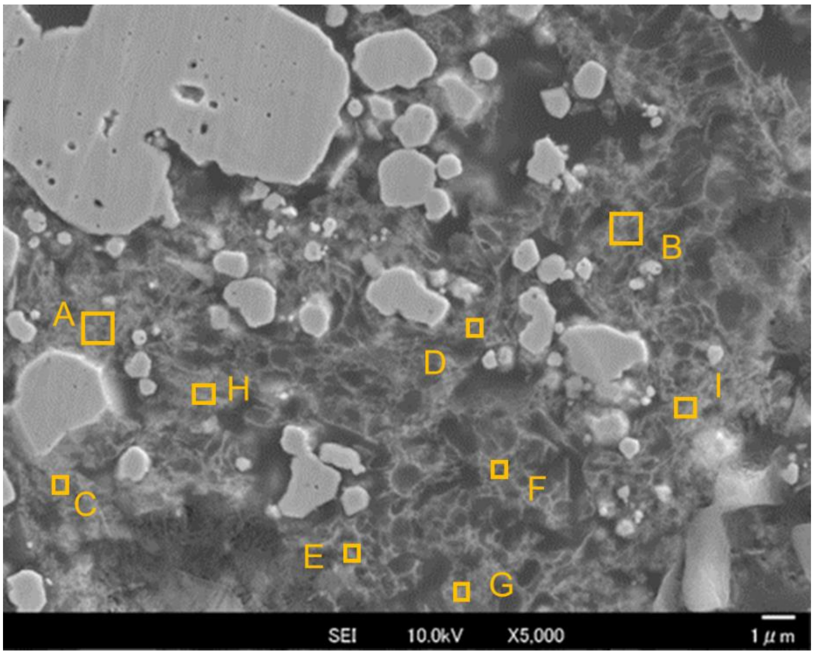

	C	O	F	Cu	Y	Y/O
A	63.7	9.6	4.1	6.4	16.2	1.688
B	79.1	6.1	4.1	0.9	9.8	1.606
C	52.3	11.3	4	8	24.4	2.159
D	70.2	6.6	4.7	2.2	16.3	2.469
E	76.8	5.7	4.3	0.2	13	2.281
F	81.5	5.7	3.3	0.1	9.3	1.632
G	70.6	7	5.1	0.3	17	2.429
H	71.8	6.8	5	0.3	16	2.353
I	1	7.1	5.2	65.7	21	2.958

Figure S13. EDX measurement points on the Cu+Y_2O_3 electrode after Li plating (top) and the EDX results (bottom).

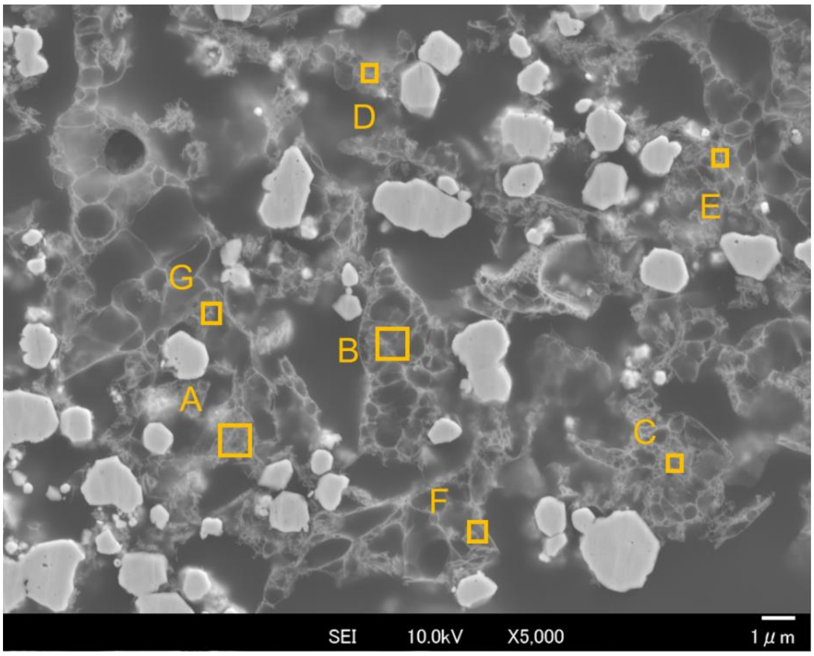

	C	O	F	Cu	Y	Y/O
A	73.8	7.1	2.4	8.1	8.7	1.225
B	83.7	6.4	1.4	3.5	5	0.781
C	84.9	5.8	1.8	0.1	7.5	1.293
D	43.9	5.3	2.6	40.1	8	1.509
E	37.7	4	1.9	51.4	4.9	1.225
F	59.5	9.5	3.4	9.6	17	1.789
G	72.1	7.6	1.7	5.8	12.8	1.684

Figure S14. EDX measurement points on the Cu+Y_2O_3 electrode before Li plating (top) and the EDX results (bottom).

3.7. ^{7}Li-NMR results

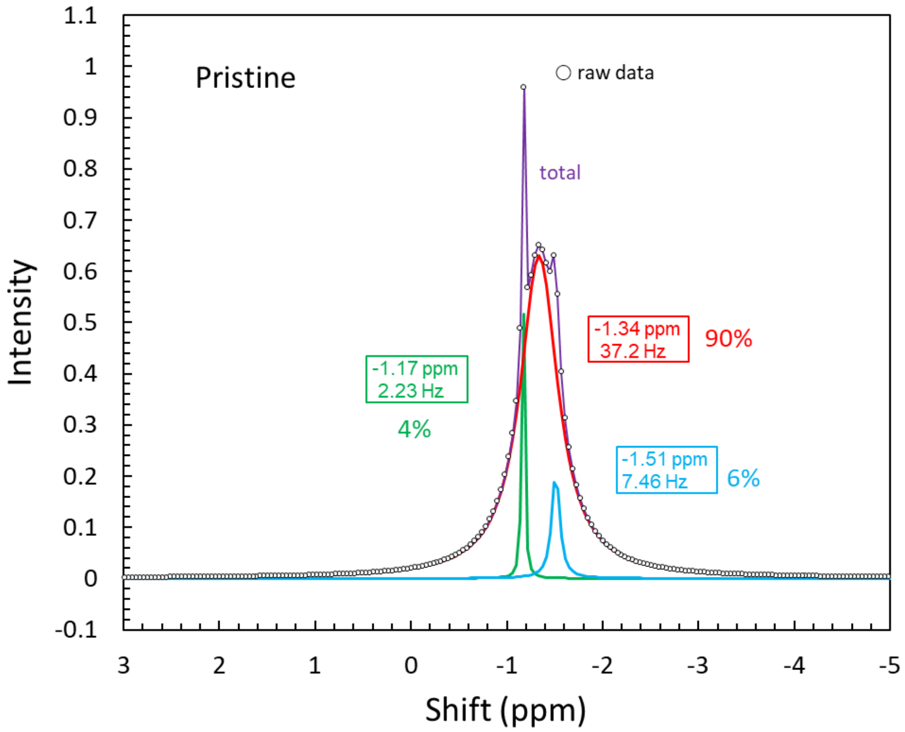

Figure S15. Waveform separation results for the region near 0 ppm in the ^{7}Li-NMR spectrum of a Cu+Y_2O_3 electrode before Li plating.
